# PP79 Efficiency Frontier Analysis Of Fostemsavir For Patients Living With Multidrug-Resistant HIV-1

**DOI:** 10.1017/S0266462325102973

**Published:** 2025-12-29

**Authors:** Lívia Nassif, Álex Martins, Bárbara Santos, Isabela Rossi, Ludmila Gargano, Francisco Acurcio, Juliana Alvares-Teodoro, Augusto Guerra

## Abstract

**Introduction:**

HIV-1 compromises the immune system, leading to immunodeficiency. Standard treatment involves the use of antiretroviral therapies; however, the development of resistance is common, highlighting the need for alternative therapeutic options. This study evaluated the therapeutic value and cost effectiveness of fostemsavir in combination with optimized background therapy (OBT), compared with OBT alone for patients living with multidrug-resistant HIV-1.

**Methods:**

A cost-effectiveness analysis was conducted to estimate the incremental cost-effectiveness ratio (ICER) of fostemsavir combined with OBT for people living with HIV-1 resistant to at least four classes of antiretroviral therapies, compared with OBT alone. The analysis was performed from the perspective of the Brazilian Unified Health System (SUS), considering a one-year treatment horizon. For OBT, it was assumed that the regimen could include the simultaneous use of up to six medications. The efficiency frontier methodology was applied to determine a proportional price based on the cost and therapeutic response benefit.

**Results:**

Fostemsavir demonstrated an incremental effectiveness of 54 percent in therapeutic response rate (viral load reduction). Over a one-year horizon, the cost-effectiveness analysis revealed a cost of BRL163,402.21 (USD27,233.70) for fostemsavir combined with OBT, compared with BRL23,271.60 (USD3,878.60) for OBT alone. The incremental cost of using fostemsavir was BRL260,127.37 (USD43,354.56) per therapeutic response. Based on the efficiency frontier approach, the incremental cost of fostemsavir should not exceed BRL19,819.83 (USD3,303.30), calculated based on the amount currently spent by the SUS plus the 54 percent additional benefit.

**Conclusions:**

The use of fostemsavir combined with OBT demonstrates a superior virologic response rate, compared with OBT. Efficiency frontier analysis suggested a maximum justified cost, providing a framework for pricing decisions. This study highlighted the importance of balancing innovation with cost effectiveness in healthcare decision-making.

